# Supplementary material for: Cancers: What Are the Costs in Relation to Disability-Adjusted Life Years? A Systematic Review and Meta-Analysis
Source: Int J Environ Res Public Health. 2022 Apr 16;19(8):4862. doi: 10.3390/ijerph19084862 (PMC9029428; doi:10.3390/ijerph19084862)

Cancers: which costs in relation to Disability-Adjusted Life Years? A Systematic Review and Meta-Analysis

SUPPLEMENTAL MATERIAL

Table S1 - Search strings

**PubMed**<br>(disability adjusted life years[MeSH Terms] OR DALY*[Title/Abstract] OR disability-adjusted[All Fields]) AND (analysis, cost[MeSH Terms] OR cost-per-daly [All Fields] OR cost*[All FIelds] OR “cost analysis”[Title/Abstract] OR economic*[All Fields]) AND (neoplasms[MeSH Terms] OR cancer*[All Fields] OR neoplasm*[All Fields] OR neoplast*[All Fields] OR oncolog*[All Fields] OR tumour*[All Fields] OR tumor*[All Fields])
**Scopus**<br>TITLE-ABS-KEY (disability AND adjusted AND life AND years OR daly* OR disability-adjusted) AND TITLE-ABS-KEY (analysis, AND cost OR cost-per-daly OR cost* OR "cost analysis" OR economic*) AND TITLE-ABS-KEY (neoplasms OR cancer* OR neoplasm* OR neoplast* OR oncolog* OR tumour* OR tumor*)
**Embase**<br>(disability adjusted life years OR DALY* OR disability-adjusted) AND (analysis, cost OR cost-per-daly OR cost* OR “cost analysis” OR economic*) AND (neoplasms OR cancer* OR neoplasm* OR neoplast* OR oncolog* OR tumour* OR tumor*)
**Web of Science**<br>(“disability adjusted life years” OR DALY* OR disability-adjusted) AND (cost-per-daly OR cost* OR "cost analysis" OR economic*) AND (cancer* OR neoplasm* OR neoplast* OR oncolog* OR tumour* OR tumor*)

Table S2 - Data collection form

Study ID	
First author, publication year	
Scope (national, international, global…)	
Country/Countries	
Assessment period (start – end)	
Aim of the study	
Study type (patient data, virtual simulation…)	
Number of patients/simulations	
Currency (and year)	
Use of age-weighting (yes/no)	
DALY discounting (yes/no)	
Discount rate	
Data source(s) for direct costs	
Data source(s) for indirect costs	
DALY source/computation method	
Corresponding country's GDP per capita (where applicable)	
Perspective (societal, governmental…)	
Neoplasm type(s)	
Direct cost estimates [95% CI]	
Indirect cost estimates [95% CI]	
Total cost estimates [95% CI]	
DALY estimates [95% CI]	
Additional notes (if any)	

TABLE S3 – Quality assessment of included studies according to the Quality of Health Economic Studies (QHES) instrument.

QHES Criteria	John et al, 2008	Oh et al, 2012	Unar-Munguia et al, 2017	Vondeling et al, 2018	Ranganathan et al, 2020	Noh et al, 2020	Neves et al, 2021
**Q1.**	7	7	7	7	7	7	7
**Q2.**	4	4	4	0	0	4	4
**Q3.**	8	8	8	8	0	8	8
**Q4.**	0	0	0	1	1	1	0
**Q5.**	0	0	9	9	0	0	0
**Q6.**	NA	NA	NA	NA	NA	NA	NA
**Q7.**	0	0	5	0	0	5	0
**Q8.**	0	7	7	7	0	0	0
**Q9.**	8	8	8	8	8	8	8
**Q10.**	6	6	6	6	6	6	6
**Q11.**	7	7	7	7	0	7	7
**Q12.**	NA	NA	NA	NA	NA	NA	NA
**Q13.**	7	7	7	7	7	7	7
**Q14.**	0	0	6	6	0	0	0
**Q15.**	8	8	8	8	8	8	8
**Q16.**	3	3	3	3	0	3	3
**TOTAL**	**58**	**65**	**85**	**77**	**37**	**64**	**58**

†*Criteria were presented in the format of "yes/no" questions, statements, or recommendations. The 16 questions are reported in the original reference of the QHES instrument.*

NA indicates that the specific questions (i.e. 6 and 12) were waived from the assessment process since no comparisons between different alternatives were conducted/expected for the included studies.

Figure S1 – Forest plot for the cost-per-DALY ratio: sensitivity analysis. Meta-analytic estimate of the average cost per DALY ascribable to cancer (and its 95% CI). This quantitative synthesis was computed and plotted as in Figure 2a, but excluding the only study not conducted in a high-income country (Unar-Munguía *et al.*).

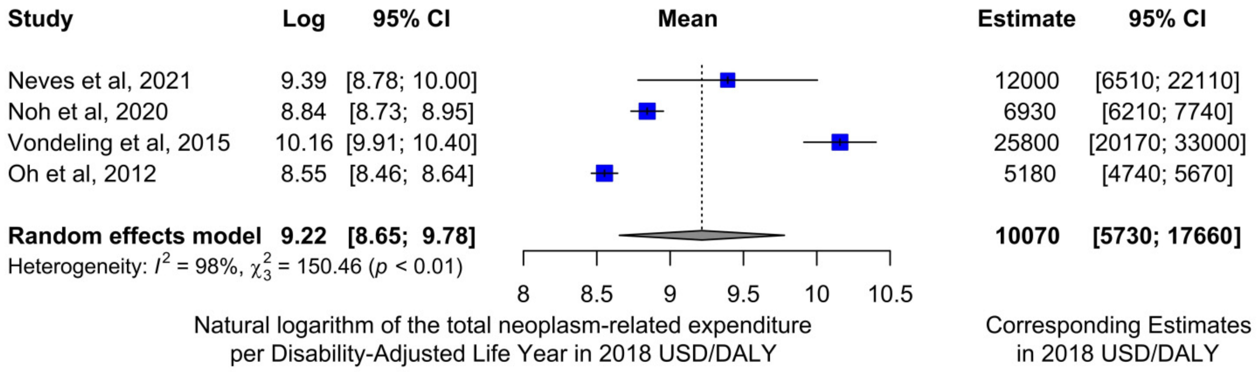

Figure S2 – Forest plot for the indirect cost-per-DALY ratio: sensitivity analysis. Meta-analytic estimate of the average indirect cost per DALY ascribable to cancer (and its 95% CI). This quantitative synthesis was computed and plotted as in Figure 3a, but excluding the only study not conducted in a high-income country (Unar-Munguía *et al.*).

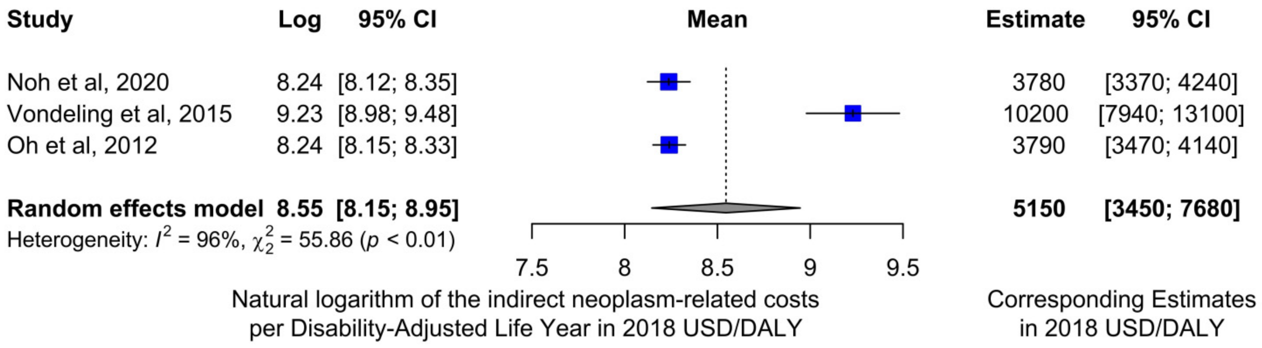

Supplement: Supplementary file 1 [file ijerph-19-04862-s001.zip › Supplementary materials 1.pdf]
